# Letter from Dr. Johnson to David Uwins, M.D. &c.

**Published:** 1830-01-01

**Authors:** 


### LIII.

### DR. JOHNSON TO DR. UWINS.

*To David Uwins, M.D. &c.*

My dear Doctor,

On returning from the Continent, a few days ago, I was much edified and amused by the perusal of your spirited manifesto of the 21st of September, in which you have honoured me with distinguished notice. I am sorry, however, my dear doctor, that you have "changed trades," and that you have become, in your own elegant phraseology, a "*Fee*-sycian instead of an Editor of Books." Consider, my dear friend, how much MEDICAL LITERATURE will lose by this vicissitude, although yourself may be the gainer! You must have reaped a golden harvest in the *fee* trade, the day you wrote your celebrated manifesto, for you plainly tell the world that you were then "upon very good terms with yourself"—and truly your lucubrations prove it! You have wisely made up for the abuse of the Lancet, and the criticisms of the Gazette, by as deep a draught of self-adulation as ever was swallowed by man!

But, my dear doctor, how do you make it out that you and I have been "private friends and public enemies" for so many years? I assure you I have never been your "public enemy"—though many people suspect that you are becoming YOUR OWN. Neither can I, my dear doctor, lay claim, conscientiously, to the sacred title of "PRIVATE FRIEND"—for, except an occasional rencontre, on publication days, in Underwood's shop, I am not aware of ever having had any intercourse or communication with you in my life. Of this I am certain—that we have never "eaten salt" together—not even *attic salt*—for you were selfish enough to hoard that up in your own "Repository." You inform us, dear Doctor, that about 15 years ago you applied to Mr. Gifford for permission to write a paper in the Quarterly Review—that the permission was granted—that an Essay "on Insanity and Mad-houses" was the result—that the Lords of the Admiralty were delighted with the paper—that Mr. Gifford was in ecstacies—that even Mr. Murray "stated to your friend Mr. Underwood that the paper was so richly deserving of something beyond the *maximum* of common payment, that he purposed asking your acceptance of another ten pound note—*which purpose, however, he never put into execution*"*—and, finally, that Mr. Underwood was so dazzled with the splendour of your "paper" in the Quarterly, that he eagerly engaged you as Editor of the Repository. The tide of Fortune, my dear Doctor, was flowing almost too fast upon you, and you know that prosperity is much more difficult to bear than adversity. I fear that it was at this time that your head became giddy by the prospect of Murray's extra ten pound note, and Underwood's appointment to the dictatorship of the medical press! It seems also that it was about this period you and I became "private friends and public enemies," without any consciousness, on my part, of this curious amico-belligerent position, for I was far from the metropolis at the time, and had little idea then of ever being elevated to the distinction of public competitor with so renowned a writer and practitioner as David Uwins, M.D. of Buckinghamshire.

"Now, Sir, simultaneously with the new-editing of the Repository, did Dr. James Johnson establish a publication, which, *if my recollection serves me right, is called the* Medico-chirurgical Review. I recollect distinctly saying to Underwood, *upon reading the first few lines of this publication*, 'you have nothing to fear—such writing and such mindless stuff as this certainly cannot do.'"

What an admirable memory, my dear David, you must possess! You not only remember the precise words of the prediction uttered *fifteen years ago*, but the very name of the worthless Journal on which the prophecy was expended! How Mr. Underwood must have chuc-

* "Should these lines reach Mr. Murray, (says the Doctor,) and he be disposed to discharge all his debts of honorable intention, he may yet hand to me, *without fear of affront*, this contemplated bonus." The keen-sighted bibliopolist, of Albemarle Street, will not fail to ask the worthy Doctor why he allowed Mr. Underwood to live 15 years without ever appealing to his testimony—and why he made the ten pound appeal directly after the amiable witness was no more! Certes this hankering after the "debt of honorable intention" is in sad keeping with the flourishing state of the fee-trade, as described by the Doctor himself.

kled at hearing this consolatory fiat from the great stipendiary writer on "Mad-houses" in the Quarterly! Pray, my dear Doctor, did Underwood also beg your acceptance of an extra ten pound note on this occasion? You hesitate to answer—well! how did the prophecy ["this certainly cannot do"] turn out? Yourself shall tell.

"It *did*, however, *do*, and it is a curious fact in the history and philosophy of periodical literature, that a Journal (the Monthly Medico-Chirurgical) was for a long time kept up without paying its expenses, which, in conjunction with Dr. Johnson, was conducted by two men (Drs. Shearman and Shirley Palmer) far, very far his superiors in every thing, while his sole and almost unassisted management of a book, which I should have thought no man of taste could have tolerated, procured fame and property to him. *Oh Templa! Oh Moses!*"

Now, my dear Doctor, the inference from the above must be, either that the medical public or yourself was in error for fifteen long years. That it was the profession which judged wrong there can be no doubt, for we have your own authority and assertion that *your* productions are characterised by great indications of talent—witness the following passage.

"When my book came out, called 'Compendium of Medical Theory and Practice,' which I assure you, Sir, is a work of *no mean merit*, Dr. J, found fault with the style, &c.!" O fie, Doctor! how came you to let this little piece of intelligence come to light in conjunction with the following eulogy on myself?

"This same Dr. Johnson is not only no writer himself but no judge of writing. Even Dr. Johnson, poor and feeble-minded as we know him well to be, could not have been *so blind to worth as his remarks on my little work would argue him to be*, were there not some other little items to be taken into account."

I am distressed beyond measure, my dear David, that you should, even now, suffer severe qualms of conscience, and that on my account. It appears that, while editor of the Medical Repository, you admitted '*a laudatory review of one of Dr. Johnson's publications*.' So so! Now this review must either have been avowed, or it was anonymous. If the *former*, I beg, my dear David, that you will unburthen your conscience, and throw the responsibility of the laudatory critique on the proper shoulders. This is the fair, open, and manly part to play with author and public. If, on the other hand, the laudatory Review was *anonymous*, your conduct in publishing it, explains fully a candid passage in your celebrated letter, "I am afraid I handed it (the Medical Repository) over to my learned friend Dr. Copland, *not much improved in value*."*

You tell us, my dear friend, that this epistle of yours to your own hebdomadal (at least you are one of the reputed editors) was penned *in the evening*, and that next morning you read it, at breakfast, to your "young wife," who pronounced it to be a very "able" production, but hinted a fear that some reprisals might be made by the objects of your pointed wit and biting sarcasms. I wonder your sensible spouse, my dear Davy, did not suggest the doubt that such lucubrations as these would excite a suspicion in the public mind either that you sacrificed rather too freely, after dinner, at the shrine of Bacchus—or that all was not right in the upper story—namely, that you were lightheaded. I, indeed, and other of your private and intimate friends, could certify that not even your neighbour Mr. Abernethy is more abhorrent of the Tuscan grape than yourself—while the whole circle of your acquaintance could vouch for

* As laudatory reviews can only proceed from the authors themselves, or from friends that are instigated by the authors or publishers, the statement of Dr. Uwins above-mentioned implies an imputation on the conduct of Dr. Johnson, which *must be cleared up*. Let Dr. Uwins state, either publickly or privately, the name of this laudatory reviewer, and Dr. Johnson pledges himself to prove that the said review was a most disinterested action—for of the writer he knows no more than he does of the "man in the iron mask," or the "man in the moon."

the solid structure of your ATTICS. But still, my dear Doctor, as the public often form their notions of a man's sense and judgment from the tenor of his writings, and as yours are by no means calculated to further the prosperity of the *fee*-trade, to which you say you have so *very recently* taken, I would seriously advise you to look after your patients in Bedford Row, rather than exhibit yourself as a literary Merry Andrew—

" Frisking beneath the burthen of three score,"

and inditing long nocturnal reveries—with your young wife's morning comments, which can only draw upon yourself and your partner the derision of that public to which you appeal.

As a " private friend " I offer you this advice, my dear Doctor, from a thorough conviction that its adoption will be beneficial to you—as a " public enemy," you may bear in mind the adage—

" Fas est et ab hoste doceri."

Your's truly,
JAMES JOHNSON.

Dec. 10th, 1829.

